# Effect of Gamma Irradiation on 2-Acetyl-1-pyrroline Content, GABA Content and Volatile Compounds of Germinated Rice (Thai Upland Rice)

**DOI:** 10.3390/plants6020018

**Published:** 2017-05-10

**Authors:** Sompong Sansenya, Yanling Hua, Saowapa Chumanee, Kannika Phasai, Chanun Sricheewin

**Affiliations:** 1 Department of Chemistry, Faculty of Science and Technology, Rajamangala University of Technology Thanyaburi, Pathum Thani 12110, Thailand; kannika_p@dss.go.th; 2 The Center for Scientific and Technological Equipment, Suranaree University of Technology, Nakhon Ratchasima 30000, Thailand; yanling@sut.ac.th; 3 Division of Chemistry, Faculty of Science and Technology, Phetchabun Rajabhat University, Mueang, Phetchabun 67000, Thailand; dr.saowapa@hotmail.com; 4 Division of Physics, Faculty of Science and Technology, Phetchabun Rajabhat University, Mueang, Phetchabun 67000, Thailand; newchanun@yahoo.com

**Keywords:** gamma irradiation, GABA, 2-acetyl-1-pyrroline, volatiles compounds, Thai upland rice

## Abstract

Aroma intensity in rice is related to the level of 2-acetyl-1-pyrroline (2AP). The accumulation of 2AP in rice has been synthesized via l-proline metabolism by inactive betaine aldehyde dehydrogenase enzyme (BADH2), which activates 2AP accumulation. Meanwhile, active BADH2 inhibits 2AP accumulation but activates γ-aminobutyric acid (GABA) accumulation. The improvement of 2AP content in rice has been reported under certain conditions, such as high salinity, water treatment, and reduction of high intensity solar exposure. In this study, we conducted the effects of gamma irradiation on 2AP content, GABA content and volatile compounds of germinated rice (Thai upland rice). Our results showed that the GABA content was highest when rice seeds germinated within a 24-h. The 2AP content of irradiated rice (germinated within a 24-h duration) was higher than non-irradiated rice for all gamma doses, particularly at 20 Gy, which showed a 23-fold higher level of 2AP than non-irradiated rice. On the other hand, the reduction of the GABA content of irradiated rice was caused by an increase in the gamma dose. At 300 Gy, irradiated rice had a GABA content approximately 2.6-fold lower than non-irradiated rice. Moreover, we observed that a reduction of volatile compounds occurred when increasing gamma dose. However, some volatile compounds appeared in the irradiated rice at gamma doses of 60 Gy, 80 Gy, 100 Gy and 300 Gy. Furthermore, we observed that the level of Octanal, which is the compound most related to aroma intensity, of irradiated rice was stronger than that of non-irradiated rice. Our results demonstrate for the first time that 2AP and GABA contents are sensitive to gamma irradiation conditions. Moreover, the results indicate that the gamma irradiation technique can be used to improve the aroma intensity of rice.

## 1. Introduction 

Rice aroma is formed by a mixture of a volatile compounds and more than 100 other compounds [1,2,3]. Among these, 2-acetyl-1-pyrroline (2AP) as characteristic compound of fragrant rice, and one of the most abundant aroma compounds found in fragrant rice is reported to be Octanal [3,4,5]. 2AP is synthesized via l-proline metabolism; the dominant betaine aldehyde dehydrogenase gene (*Badh2*) inhibits 2AP synthesis, while the recessive betaine aldehyde dehydrogenase gene (*badh2*) activates 2AP accumulation [6,7]. The recessive *badh2* gene encoding the inactive betaine aldehyde dehydrogenase (BADH2) catalyzes the substrate γ-aminobutyraldehyde (GABald) to Δ^1^-pyrroline, and finally acetylates 2AP. Meanwhile, the dominant *Badh2* gene encoding the active BADH2 catalyzes the substrate GABald to form γ-aminobutyric acid (GABA) [7,8]. GABA accumulation in plants has been reported via two pathways. In the first, the active BADH2 enzyme catalyzes GABald to form GABA [7]. In the second, the production of GABA occurs through the GABA shunt [9]. Moreover, 2AP accumulation is affected by several environment factors. Take, for example, Khao Dawk Mali 105, a famous Thai aromatic rice variety. Samples of this variety of rice samples were collected from an area under drought conditions, and 2AP accumulation was higher in these samples than that in rice samples collected from an area under non-drought conditions [10]. Recently, the increase of 2AP content has been shown to respond to salt stress and, in addition, the expression of *Badh2* gene has shown a correlation with salt stress [11,12,13]. One more environment factor to take into consideration is shading treatment. Mo et al. [5] found that 2AP accumulation was significantly increased in rice samples under all shading conditions. GABA accumulation in rice plants has also been shown to respond to biotic and abiotic stress [14,15,16]; GABA accumulation in rice is affected by several factors, such as salt concentration, duration of rice seed germination, rice genotype and rice cultivar [13,17,18,19]. While the effect of both 2AP and GABA accumulation in rice has been reported to be affected by shading treatment, meaning the reduction of exposure to solar intensity, one study found that 2AP and GABA content in rice significantly increased when placed under all types of shading treatment [5]. According to another report, the relationship between 2AP and GABA content responds to salt treatment during growth stages. The results showed that salt treatment activates 2AP content in leaves, but does not affect GABA content [13].

It is considered well-known that rice is a major food source around the world. In addition, the aroma of fragrant rice is considered to be a highly important feature in the rice market. As a previous researcher reported, several techniques have been used to improve the aroma intensity of rice, such as salt stress, drought stress and the reduction of solar intensity [5,10,13]. In the current research, we conducted one such technique to improve rice aroma; we employed gamma ray irradiation. Gamma ray is a type of ionizing radiation, and this technique can be used to induce plant mutation. Gamma ray irradiation can produce reactive oxygen species (ROS), which causes DNA damage and creates mutations in plants [20,21]. In rice, gamma ray irradiation is mostly used for the improvement of rice grain quality as well as to affect the growth rate of rice. As previously reported, gamma ray irradiation causes higher tocopherol content in rice mutant lines than in the control rice [22]. Sasikala and Kalaiyarasi [23] found that gamma ray irradiation affects seed germination, as well as shoot and root lengths of rice. Moreover, Morita et al. [24] found that rice has base deletions and base substitution when treated with gamma ray irradiation. Recently, Sansenya et al. [25] showed that when seeds of the upland rice variety (Thai black glutinous rice) were gamma-irradiated, the resultant 2AP content of shoots was stimulated under the gamma ray treatment. Moreover, gamma ray irradiation also caused the increase of 2AP content in rice grains, but also caused a reduction of the grain yield. While this study was to determine the effect of gamma irradiation on 2AP content and GABA content, and we also researched the changes in volatile compounds in irradiated rice compared with non-irradiated rice, in order to improve the aroma intensity in germinated rice (Thai upland rice).

## 2. Results

### 2.1. The Effect of Germination Time on the GABA Content of Thai Upland Rice 

**Figure 1:**
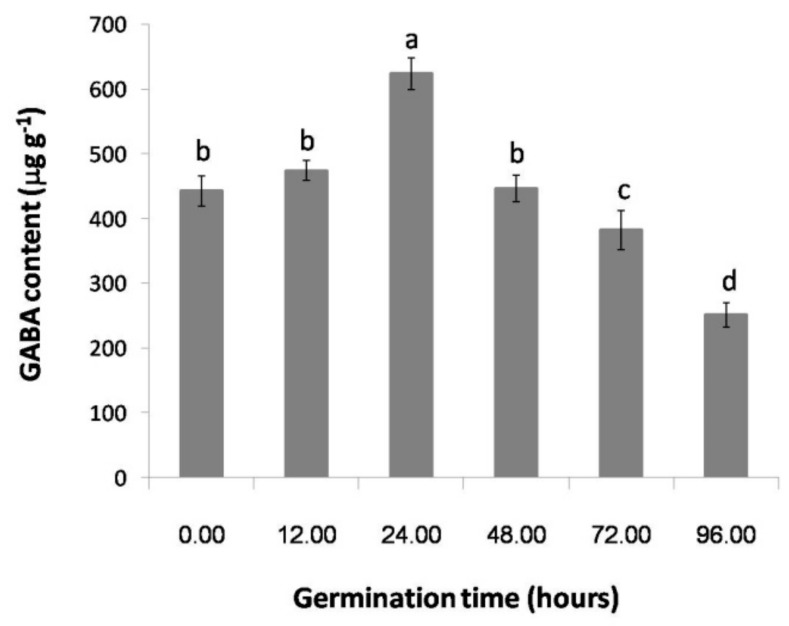
The GABA content at different germination times. The error bars indicate the standard deviation of means (*n* = 3); the same letters indicate no significant difference (Duncan, *p* > 0.05).

Figure 1 shows that the GABA content of Thai upland rice increased after 12 h when compared with 0 h (no germination time), and the highest GABA content of 623.67 ± 24.19 μg g^−1^ was observed at 24 h. After the highest value, the GABA content decreased continuously from 48 h to 96 h duration of germination time. The optimized germination time that produced the highest GABA content was further used in experiments to test the effect of gamma irradiation on GABA content, 2AP content and volatile compounds.

### 2.2. The Effect of Gamma Irradiation on the GABA and 2AP Contents of Non-Irradiated and Irradiated Rice 

**Figure 2:**
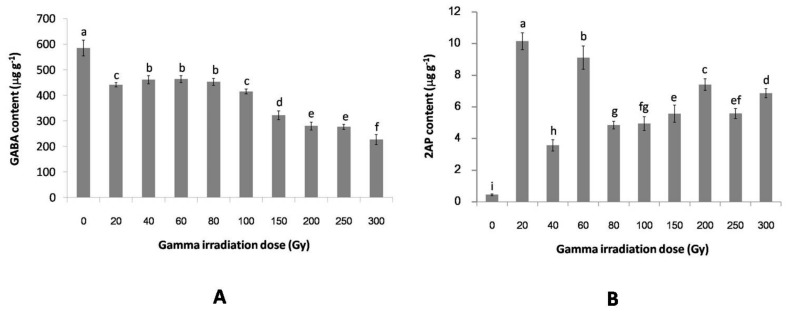
The comparison of GABA content (**A**) and 2AP content (**B**) in different gamma doses of rice germinated for a duration of 24 h. The error bars indicate the standard deviation of means (*n* = 3), the same letters indicate no significant difference (Duncan, *p* > 0.05).

The germinated rice at 24 h (non-irradiated rice and irradiated rice) was used to observe the effect of gamma irradiation on GABA and 2AP levels. Our observation shows that the GABA content of the irradiated rice significantly (*p* < 0.05) decreased with all gamma doses compared with the non-irradiated rice. At the gamma dose of ≥200 Gy, the GABA content of the irradiated rice decreased approximately twice as much as in the case of the non-irradiated rice. On the other hand, the 2AP content of the irradiated rice increased with all gamma doses, and the increase was approximately eight-fold to 23-fold higher than those of the non-irradiated rice. Moreover, the highest 2AP level of 10.28 ± 0.53 μg g^−1^ was obtained at 20 Gy (Figure 2).

### 2.3. The Effect of Gamma Irradiation on Volatile Compounds of Non-Irradiated and Irradiated Rice 

Table 1 shows all volatile compounds identified from the irradiated rice and the non-irradiated rice (germination time of 24 h). Thirty-four volatile compounds were obtained from the non-irradiated rice (0 Gy), and the same amount were also obtained from the irradiated rice treated with 20 Gy and 40 Gy. For other gamma doses, 33 volatile compounds were obtained from the irradiated rice treated with 60 Gy and 80 Gy, respectively. Thirty-one volatile compounds were recorded in the irradiated rice treated with 100 Gy, 150 Gy and 200 Gy. Thirty and 28 volatile compounds were recorded in the irradiated rice treated with 250 Gy and 300 Gy, respectively. Among the detected compounds, the characteristic fragrant rice compound 2AP was identified in both the irradiated and non-irradiated rice. Other aroma compounds were also identified in all of the gamma doses (including of 0 Gy), such as Hexanal, Undecane, Dodecane, 2-Pentylfuran, Octanal, Tridecane, (*Z*)-2-Heptenal, Nonanal, Tetradecane, 3-Octen-2-one, 1-Octen-3-ol, Benzaldehyde, Octylformate, *n*-Nonylcyclohexane, 2-Butyl-2-octenal, Undecane, Methyl *N*-hydroxybenzenecarboximidate, 2-Ethyl-3-hydroxyhexyl 2-methylpropanoate, Benzyl alcohol and 2,3-Dihydrobenzofuran. Furthermore, some volatile compounds were identified in some gamma doses of irradiated rice. 1-Butyl-2-pentyl-cyclopentane, 1-Hexanol, Isobutyl tridecyl carbonate, Cyclotetradecane and 3-Methyl-Pentadecanewere only recorded in the rice treated with 60 Gy. Hexyl butanoate and Nonanoic acid were identified in the rice treated with 80 Gy. Only *n*-Hexadecanoic acid and 5-Methyl-2-hexanone were identified in the rice treated with 100 Gy and 300 Gy, respectively.

## 3. Discussion 

We reported that GABA accumulation in germinated rice was detected at 12 h duration, and the highest content was about 1.4-fold that obtained from the rice germinated for 24 h compared with 0 h (Figure 1). The accumulation of GABA after the highest value at a 24-h germination duration rapidly decreased, and the lowest content occurring at 96 h was 2.5-fold lower than that at 24 h. As previously reported, Karladee and Suriyong [26] found for that almost 21 rice varieties, including the upland rice varieties, the highest GABA accumulation was obtained from rice germinated for 24 h. A slight difference was observed from a study of Indica and Japonica rice, in which the highest GABA accumulations were detected at 36 h for both rice genotypes. At the peak of GABA accumulation, Indica rice seems to have a higher GABA accumulation than Japonica rice, while the glutamate decarboxylase activity (GAD) of both rice genotypes appeared in the highest value at 24 h [19].

The accumulation of GABA and 2AP in plants is synthesized via l-proline metabolism, and acts as active BADH2 catalyzing the conversion of GAbald to GABA, while the inactive BADH2 cyclisation of GAbald forms Δ^1^-pyrroline and finally the addition of an acetyl group forms 2AP [6,7]. In plants, other pathways for GABA accumulation may occur via the GABA shunt [9]. Our results show that the 2AP content of the non-irradiated rice (0 Gy) was 23-fold lower than the highest 2AP content of the irradiated rice (20 Gy). On the other hand, the GABA content of the non-irradiated rice (0 Gy) was 2.6-fold higher than the lowest GABA content of the irradiated rice (300 Gy). Moreover, the 2AP content of the irradiated rice increased with all gamma doses compared with the non-irradiated rice, while the GABA content of the irradiated rice decreased with all gamma doses compared with the non-irradiated rice. Recently, we reported the effect of gamma irradiation on the 2AP content and plant growth of Thai black glutinous rice. The present data indicated that the 2AP content of the irradiated rice shoots were higher than those of the rice shoots of non-irradiated rice. Also, the 2AP content of the rice grains was stimulated by gamma irradiation, and at high gamma doses the 2AP content was higher than that of the low gamma doses [25]. A previous researcher reported that high gamma doses cause DNA damage, inhibition of cell cycle, base deletions and base substitution, decrease of soluble protein, decrease of photosynthetic pigments and inhibition of plant growth [21,24,25,27,28,29]. These effects by gamma irradiation have hypothesized the inhibition of GABA accumulation in both pathways (l-proline metabolism and GABA shunt), while the inhibition of the GABA content via l-proline metabolism may activate the synthesis of 2AP content. This suggests that the accumulation of both GABA and 2AP occurs via l-proline metabolism. Furthermore, our results showed larger changes in 2AP contents than in GABA contents between non-irradiated and irradiated rice; this result may be because GABA is synthesized via two pathways and while 2AP is synthesized only via l-proline metabolism.

Our study shows that the volatile compounds of irradiated rice decreased with the increase of the gamma dose (Table 1 and Table 2). A previous researcher reported that the increase of the gamma dose caused a decrease of biochemical compounds [29]; this may explain the decrease in volatile compounds in our study. The volatile compounds 2AP and octanal have been abundantly reported in fragrant rice [3,4,5,30]. Our results show that these compounds were detected for all gamma doses, and the 2AP content of the irradiated rice was at a higher level than that of the non-irradiated rice, also the level of Octanal in the irradiated rice seemed to be higher than that of the non-irradiated rice (Table 1 and Table 2). Concerning Octanal, this compound is related to sweet and odor-active compounds [31]. Therefore, our results suggest that the sweet odor of the irradiated rice seemed to be higher than that of non-irradiated rice. 2AP was reported to be the key compound of fragrant rice, and its concentration is directly related to the sensory intensities of the rice sample’s aroma [32]. Our results indicate that high sensory intensities in the irradiated rice was stronger than the non-irradiated rice due to the 2AP contents of the irradiated rice being higher than the non-irradiated rice. Some volatile compounds were observed in some gamma doses of irradiated rice. 1-Hexanol and Nonanoic acid were identified only in rice treated with 60 Gy and 80 Gy, respectively. A previous researcher found that the content of these compounds in fragrant rice were higher than in non-fragrant rice, while 1-Hexanol is one of the products derived from either the oxidation or degradation of lipids [33,34,35,36]. Furthermore, Hexadecanoic acid was only reported in irradiated rice treated with 100 Gy, which is related to the lipid structure and function of the cell membrane [37,38]. Our results suggest that this compound is associated with the cell membrane of irradiated rice in the function of gamma irradiation tolerance.

In conclusion, we documented that the significant increase of GABA content in rice found at a 24-h germination duration was higher than that at 0 h of germination. Furthermore, The 2AP content of irradiated rice derived from the 24-h germination time was higher than that of non-irradiated rice. On the other hand, the GABA content of irradiated rice was lower than that of non-irradiated rice. Our results indicate that gamma irradiation affects both L-poline metabolism and GABA shunt. The volatile compounds of irradiated rice decreased when the gamma dose increased, in comparison to non-irradiated rice. Some volatile compounds were detected the irradiated rice treated with some gamma doses. Our results indicate that gamma irradiation affects the metabolism of that compound such as 1-Hexanol, Nonanoic acid and Hexadecanoic acid.

## 4. Materials and Methods

### 4.1. Plant Material 

Rice grains (Thai upland rice) were obtained from a rice field in Petchabun province, Thailand. The rice grain samples of 50 grams were soaked with 0.1% NaClO for 30 min and then rinsed with distilled water for sterilization, and finally dried in a hot-air oven to decrease the moisture content to <13%.

### 4.2. Germination Time and GABA Content Quantification

For the germination time, 15 grams of rice grains were soaked with distilled water for 24 h. They was then germinated on germinating paper moistened by distilled water, with the germination experiment at 12, 24, 48, 72 and 96 h. The germination samples were sprayed with distilled water every 12 h. The germinated rice was stopped by the end of that hour condition with water steaming for 2 min, then roasting in a hot-air oven at 60 °C for 6 h. The seed coat of germinated rice was taken out before being homogenized with a Cryo Mill (Retsch, Haan, Germany). The samples were kept at 4 °C until the GABA content was correctly quantified.

For GABA quantification using the modified method of Zhang et al. [19], 2 grams of each sample was weighed into a 15-mL plastic tube and dissolved with 5 mL of deionized water, and then extracted for 30 min, followed by centrifugation at 10,000 rpm for 15 min. The solution on top was filtered by using a Syringe Filter (0.45 μm), and then 0.5 mL of the filtered sample mixed with 0.2 mL of 0.2 M borate buffer pH 9.0, 1 mL of 6% phenol reagent and 0.4 mL of 9% NaClO. Then the solution was mixed and shaken thoroughly. Further, the mixture was boiled for 10 min, and then cooled in cooling bath for 20 min or until a blue color appeared. The samples for GABA quantification were determined by spectrophotometry at a wavelength of 645 nm.

The GABA content of each sample was quantified by comparing the value of the absorption with the content curve of standard GABA y = 1.28x − 0.024 (*R^2^* = 0.994). For standard GABA (final concentration between 0.00, 0.01, 0.03, 0.05, 0.07, 0.09 and 0.10 mg L^−1^), the reaction was mixed with 0.2 M borate buffer pH 9.0, 1 mL of 6% phenol reagent and 0.4 mL of 9% NaClO, and then measured with spectrophotometry at a wavelength of 645 nm.

The germination time that produced the highest GABA content was further used to test the effect of gamma irradiation on GABA content, 2AP content and volatile compounds analysis of rice.

### 4.3. Gamma Irradiation 

For gamma irradiation, the sterilized rice grains were packed in a polyethylene bag, then exposed to gamma ray irradiation of 0 Gy (mean; non-irradiated rice) and 20, 40, 60, 80, 100, 150, 200, 250 and 300 Gy (mean; irradiated rice) with a dose rate of 3 Gy min^−1^ by using ^137^Cs as a gamma source at room temperature. The gamma irradiation treatments were provided by Gamma Irradiation Service and Nuclear Technology Research Center, Faculty of Science, Kasetsart University, Thailand.

### 4.4. The Effect of Gamma Irradiation on GABA Content, 2AP Content and Volatile Compounds 

The irradiated rice seeds (0, 20, 40, 60, 80, 100, 150, 200, 250 and 300 Gy) were soaked with distilled water for 24 h. The germinated rice was produced with germinating paper moistened by distilled water, at the germination time that produced the highest GABA content (24 h). The experiments were sprayed with distilled water every 12 h. The germination experiments were stopped at the end of the hour that produced the highest GABA content. The sample processing was the same as the experiment above (4.2). The samples were kept at 4 °C until GABA quantification, 2AP quantification and volatile compounds analysis.

### 4.5. 2AP Quantification with Gas Chromatography-Mass Spectrometry (GC-MS)

For 2AP extraction, the germinated rice samples (non-irradiated and irradiated rice) were homogenized with liquid nitrogen cooling. Then, the 2AP content was extracted by the methods of Sansenya et al. [25].

For 2AP quantification, the 2AP standard was purchased from BOC Sciences (New York, NY, USA) with a purity of 95%. The 2AP content was analyzed by using a Bruker gas chromatograph 450-GC and TQ mass spectrometer 320-MS. Also, 0.01 mg mL^−1^ of trimethyl pyridine (TMP) was used as internal standard in this analysis. The GC-MS conditions for 2AP quantification was analyzed by the method of Sansenya et al. [25]. Further the amounts of 2AP were calculated from its calibration curve.

### 4.6. Analysis of Volatile Compounds

About 1 g of homogeneous germinated rice samples (non-irradiated rice and irradiated rice) were weighed into a vial with 20 ml of headspace and capped. Each sample was pre-heated at 80 °C for 50 min, and then a SPME fiber (50/30 μm DVB/CAR/PDMS, SUPELCO, Bellefonte, PA, USA) was used to extract volatile compounds for 30 min. The fiber was desorbed in GC injector port at 250 °C for 5 min. Separation of the desorbed volatile compounds was achieved by gas chromatography-mass spectrometry (Agilent 7890A GC-7000 Mass Triple Quad) equipped with a capillary column (DB-WAX, 60 m × 0.25 mm × 0.25 μm, J&W Scientific, Folsom, CA, USA) and a quadrupole mass detector. The injector was operated in splitless mode for 3 min then switched to split mode. Helium gas was used as the carrier gas with a constant flow rate of 1.0 mL min^−1^. The GC oven temperature was started at 30 °C for 10 min, and increased to 160 °C at 3 °C min^−1^, then increased to 230 °C at 8 °C min^−1^ and was held for 8 min. The mass spectrometer was used in the electron ionization mode with the ion source temperature set at 230 °C and the ionization energy set at 70 eV. The scan mode was used and the scan range was 30 to 500 *m*/*z*. Identification of volatile compound was performed by comparing mass spectra with NIST mass spectral libraries (National Institute of Standards, 2011 version). The content of volatile compounds was calculated from the peak areas.

### 4.7. Statistical Analysis

Statistics and data analysis were expressed as the mean ± standard deviation (mean ± SD). All computations were carried out using the SPSS version 22.0 software for Windows (IBM customer no.441840, Phetchabun Rajabhat University, Phetchabun, Thailand). The statistical significance of differences among experimental groups was evaluated by one-way analysis of variance (ANOVA). When the ANOVA revealed significant between-group differences, a post hoc analysis was performed using Duncan’s multiple-range test comparisons. The significance level was set at *p* < 0.05.

## Figures and Tables

**Table 1:** The volatile compounds of non-irradiated rice (0 Gy) and irradiated rice (20 Gy to 300 Gy) identified from rice germinated for a duration of 24 h.

RT (min)	Volatile Compounds	0 (Gy)	20 (Gy)	40 (Gy)	60 (Gy)	80 (Gy)	100 (Gy)	150 (Gy)	200 (Gy)	250 (Gy)	300 (Gy)
21.62	Hexanal	+	+	+	+	+	+	+	+	+	+
22.37	Undecane	+	+	+	+	+	+	+	+	+	+
27.58	5-Methyl-2-hexanone	−	−	−	−	−	−	−	−	−	+
27.64	Heptanal	−	−	−	+	−	+	+	+	+	+
28.52	Dodecane	+	+	+	+	+	+	+	+	+	+
29.56	Butyl butanoate	−	−	−	−	−	−	+	+	−	−
30.17	2-Pentylfuran	+	+	+	+	+	+	+	+	+	+
33.13	Octanal	+	+	+	+	+	+	+	+	+	+
33.74	Tridecane	+	+	+	+	+	+	+	+	+	+
34.20	1-Butyl-2-pentyl-cyclopentane	−	−	−	+	−	−	−	−	−	−
34.84	(*Z*)-2-Heptenal	+	+	+	+	+	+	+	+	+	+
35.57	2-Acetyl-1-pyrroline	+	+	+	+	+	+	+	+	+	+
35.73	Cyclododecane	−	−	−	+	−	−	−	−	−	+
36.43	1-Hexanol	−	−	−	+	−	−	−	−	−	−
37.99	Heptylcyclohexane	−	−	−	+	−	−	+	+	+	+
38.16	Nonanal	+	+	+	+	+	+	+	+	+	+
38.30	Tetradecane	+	+	+	+	+	+	+	+	+	+
38.83	3-Octen-2-one	+	+	+	+	+	+	+	+	+	+
39.18	Hexyl butanoate	−	−	−	−	+	−	−	−	−	−
39.79	8-Methyloctahydrocoumarin	+	+	+	−	+	+	−	−	+	+
40.31	*n*-Pentadecanol	+	+	+	+	+	−	−	−	−	−
40.75	1-Octen-3-ol	+	+	+	+	+	+	+	+	+	+
41.06	2-Ethyl-1-decanol	−	−	−	−	+	+	+	+	−	−
41.07	Isobutyl tridecyl carbonate	−	−	−	+	−	−	−	−	−	−
41.08	2-Butoxyethyl acetate	+	+	+	−	−	−	−	−	−	−
42.57	1-Hexadecanol	+	+	+	−	+	+	+	+	+	+
42.58	Cyclotetradecane	−	−	−	+	−	−	−	−	−	−
42.76	4-(1-Acetyl-cyclopentyl)-but-3-en-2-one	+	+	+	−	−	−	−	−	−	−
43.87	Benzaldehyde	+	+	+	+	+	+	+	+	+	+
44.62	*n*-Heptadecanol-1	+	+	+	−	−	+	+	+	+	+
44.84	4-Ethyl-Tetradecane	+	+	+	−	+	−	−	−	−	−
44.85	3-Methyl-Pentadecane	−	−	−	+	−	−	−	−	−	−
45.37	Octylformate	+	+	+	+	+	+	+	+	+	+
46.19	[*S*-(R*,R*)]-2,3-Butanediol,	+	+	+	−	+	−	−	−	+	−
46.68	5,5-Diethyltridecane	+	+	+	+	+	+	+	+	+	−
47.14	*n*-Nonylcyclohexane	+	+	+	+	+	+	+	+	+	+
48.02	2-(2-ethoxyethoxy)-Ethanol	+	+	+	−	+	+	+	+	−	−
49.74	2-Butyl-2-Octenal	+	+	+	+	+	+	+	+	+	+
51.03	Undecane	+	+	+	+	+	+	+	+	+	+
53.03	Methyl *N*-hydroxybenzenecarboximidate	+	+	+	+	+	+	+	+	+	+
57.03	2-Ethyl-3-hydroxyhexyl 2-methylpropanoate	+	+	+	+	+	+	+	+	+	+
57.32	Benzyl alcohol	+	+	+	+	+	+	+	+	+	+
57.63	2,2,4-Trimethyl-1,3-pentanediol diisobutyrate	+	+	+	+	+	+	+	+	+	−
58.31	Butylatedhydroxytoluene	−	−	−	+	−	−	−	−	−	−
61.15	Phenol	−	−	−	−	+	+	+	+	+	−
65.36	Nonanoic acid	−	−	−	−	+	−	−	−	−	−
62.58	Octanoic acid	+	+	+	−	−	−	−	−	−	−
64.37	6,10,14-Trimethylpentadecan-2-one	+	+	+	−	−	−	−	−	−	−
70.77	2,3-Dihydrobenzofuran	+	+	+	+	+	+	+	+	+	+
74.13	*n*-Hexadecanoic acid	−	−	−	−	−	+	−	−	−	−

RT: retention time, −: absence of compound, +: presence of compound.

**Table 2:** The different levels (peak area (%)^2^) of 2AP and Octanal of non-irradiated rice (0 Gy) and irradiated rice (20 Gy to 300 Gy) identified from rice germinated for a duration of 24 h.

No.	Compound		Peak Area (%)^2^								
		0 (Gy)	20 (Gy)	40 (Gy)	60 (Gy)	80 (Gy)	100 (Gy)	150 (Gy)	200 (Gy)	250 (Gy)	300 (Gy)
1	2-Acetyl-1-pyrroline	2.76	2.98	2.98	3.42	3.22	4.83	3.36	3.52	3.74	3.72
2	Octanal	2.72	3.08	2.78	1.75	3.32	2.13	2.54	1.92	2.87	4.10
